# Knowledge, attitude and practices towards surgical wound care and healing among the public in the Jazan Region, Saudi Arabia

**DOI:** 10.1097/MD.0000000000036776

**Published:** 2023-12-22

**Authors:** Hassan Mashbari, Sulaiman Hamdi, Hussam Darraj, Mohammed Awaf, Shaden Zaalah, Faisal Hakami, Khalid M. Hakami, Essam Alhazmi, Layla Al khairat, Shatha A. Hakami, Amani Aburasain, Ibrahim Ali I. Hakami, Abdulaziz A. Arishi

**Affiliations:** a Department of Surgery, Faculty of Medicine, Jazan University, Jazan, Saudi Arabia; b Jazan University, Faculty of Medicine, Jazan, Saudi Arabia.

**Keywords:** healing, Jazan, Saudi Arabia, surgical sites infection, wound

## Abstract

The purpose of this research was to evaluate how much the people in the Jazan region know about the care and healing of surgical wounds. Proper care of surgical wounds is very important to achieve the best treatment outcomes and to avoid negative consequences. However, factors like obesity, diabetes, and certain medications can impair wound healing, with surgical site infections being a major problem in the healthcare system. Therefore, this study aimed to determine public awareness and perceptions of surgical wound care to help improve education and raise awareness of the importance of proper wound care for better results. We run an observational cross-sectional study among adults above 18 years in the Jazan region. An online self-administered questionnaire was used in the collection of data. Simple random sampling was the used technique and 384 participants were calculated. The study used Statistical Package for the Social Sciences (SPSS) for data analysis and employed descriptive statistics, independent *t* test, Analysis of Variance (ANOVA), Pearson’s correlation, and multivariate logistic regression to identify factors associated with knowledge of surgical site infection and wound care. This study analyzed 599 participants’ knowledge, attitude, and practice about surgical site infection and wound management. While participants had a strong general understanding of surgical wounds, only 17% had a high degree of knowledge about surgical site infection and wound management. Medical students had the highest degree of knowledge, and being a medical student was the only significant predictor of having a high level of knowledge about surgical site infection (SSI) and wound care. The study emphasizes the necessity of enhanced patient education and investment in medical education quality. The participants in this study had high overall knowledge regarding surgical wounds but lacked particular knowledge concerning surgical site infection and wound management. Medical education was discovered to be a strong predictor of having a high level of knowledge about surgical site infection and wound management. Healthcare professionals should take the lead in giving accurate and reliable information regarding wound care techniques to patients, and legislators should invest in enhancing medical education quality.

## 1. Introduction

A wound is any damage or break in the surface or integrity of the skin. When an injury occurs, such as after surgery, a cut, chemicals, heat/cold, pressure, or as a result of any illness, such as carcinomas or leg ulcers, the underlying connective tissues may be damaged as well.^[1]^A surgical wound is defined by the World Health Organization as “a wound created when an incision is made with a scalpel or other sharp cutting device and then closed in the operating room by suture, staple, adhesive tape, or glue and resulting in close approximation to the skin edges.” There are 4 types of surgical wounds: clean (uninfected operative wound – without inflammation), clean-contaminated (an incision that allows access to the pulmonary, gastrointestinal, or genitourinary tract without leakage), contaminated (refers to open, fresh, accidental wounds due to a major break in sterile technique or gross spillage from the gastrointestinal tract, or an incision with acute, non-purulent inflammation), dirty or infected (those that involve existing clinical infection or perforated viscera or acute inflammation with pus).^[2]^According to a 2018 retrospective analysis of Medicare beneficiaries, 8.2 million people were affected by wounds with or without infection. Medicare’s cost estimates for acute and chronic wound treatments ranged between $28.1 billion and $96.8 billion. Most expenses were incurred by surgical wounds followed by diabetic foot ulcers, with an increase in costs associated with outpatient wound care compared to inpatient care. Increasing health care costs, an aging population, recognition of increasingly difficult-to-treat infection threats such as biofilms, and an accelerating prevalence of diabetes and obesity worldwide contribute to chronic wounds being a major clinical, social, and economic challenge worldwide.^[3]^Surgical wounds are the most common wounds addressed in acute care settings, and they’re linked to a range of complications like hemorrhage and dehiscence. Surgical site infections (SSIs), on the other hand, are the most common complication – and the most avoidable hospital acquired infection.^[4]^ Any purulent discharge from a closed surgical incision, with signs of inflammation of the surrounding tissue should be considered as wound infection, and the infection can happens within 30 days of operation. There are factors that susceptibility of wound infection. These factors include preexisting illness, length of operation, wound class, and wound contamination. Other factors include intrinsic extrinsic factors Famakinwa et al (2014). And intrinsic factors that include advanced age, malnutrition, metabolic diseases, smoking, obesity, hypoxia, immunosuppression, and length of preoperative. Pre-operative skin preparation and skin antiseptics, antibiotic prophylaxis, inadequate sterilization of surgical instruments, surgical drains, surgical hands scrubs, and dressing techniques formed the extrinsic factors. The major problem is not the lack of effective surgical precautions and evidence-based guidelines, but possession of knowledge, development of the right attitude and intention to carry out these guidelines to prevent SSIs.^[5,6]^Inadequate wound care in critically ill patients resulted in bacteremia or sepsis following surgery, as well as a higher death rate.^[7,8]^As a result, critical patient wound management is a vital component of critical nursing care, and health practitioners must pay close attention to wound care. Wound healing is a multi-step process that starts with an injury and progresses through a sequence of physiologic reactions and responses that affect the wound’s potential to heal. Depending on the type of the wound, the process typically proceeds in a coordinated manner that takes time. In humans, tissue and organ healing following surgery is nearly exclusively accomplished by scar tissue replacement and regeneration.^[9]^ It is essential to identify the factors that impair wound healing with the underlying pathophysiology that interferes with the healing response to tissue injury preemptively. These factors include co-morbid conditions (diabetes, obesity, protein energy malnutrition), medications (steroids, non-steroidal anti-inflammatory drugs, antirejection medications), oncology interventions (radiation, chemotherapy), and lifestyle habits (smoking, alcohol consumption).^[10]^

Wound care is essential for optimizing the outcome of practitioners’ medical and operational treatment, as well as avoiding potentially unpleasant outcomes. As a result, wound care knowledge is the cornerstone for achieving the targeted care goals for injured patients and those who care for them. Thus, our purpose in conducting this study is to assess the knowledge, attitudes, and practices of the general public that doesn’t include health-care workers toward surgical wound care and healing to determine the perception and common practices and factors that may influence wound healing, which could in turn help improve the wound care practices of the public in Jazan region, Saudi Arabia.

## 2. Materials and methods

### 2.1. Study design and participants

This is a cross-sectional observational study aimed to assess the adult knowledge, attitude and practices toward surgical wound and healing. The study has been conducted among adults over the age of 18 years in the Jazan region, which is located in the southwest of Saudi Arabia, north of Yemen, its population consists of 1.6 million people. The study population include all adult (male, female) aged above 18 years old at Jazan region who meet the inclusion criteria: adult above 18 years old, lives in the Jazan region population, and complete the survey. We used a validated questionnaire. The data has been collected using a web Arabic language self-administered questionnaire. The link of the questionnaire has been distributed to the targeted population. The gathered data are anonymous, and neither data allowing identification of respondents nor sensitive data were collected. The questionnaire is divided into 4 sections; each of them contains questions, and each question is designed to measure a specific item: demographic characteristics of participants, measuring the extent of knowledge about the causes and signs of complications in post-operative wounds, attitudes about wounds and wound care, and practices towards wounds after surgery. A pilot study including 10% of the required sample size (39 individuals, respectively) has been conducted to evaluate the reliability and validity of the survey used for data collection. Depending on the result of the pilot study, certain improvements and reordering of some questions have been taken into consideration. The result of the pilot study was not included in the analysis of final data.

### 2.2. Sample size and statistical analysis

Sample size for this study was calculated as 384 participants using the sample size formula for studies organized according to a cross-sectional study design. The study uses the parameters of *P* = 50% to provide the maximum sample size, 95% confidence interval, and error not more than 5%. Also, the study assumed a non-response rate of 25%. The sampling design used was simple random sampling technique. Data analysis was performed using Statistical Package for the Social Sciences, SPSS 23rd version (IBM Corp., Armonk, NY. Frequency and percentages were used to display categorical variables. Minimum, maximum, mean, and standard deviation were all used to present numerical variables. Independent *t* test and Analysis of Variance (ANOVA) test were used to test for factors associated with knowledge toward surgical site infection and wound care. ANOVA test was followed by post hoc test to determine where the exact difference between subgroups exist. Pearson’s correlation was used to test for correlation between age and knowledge score. Multivariate logistic regression was used to determine the predictors for high knowledge level toward surgical site infection and wound care. The logistic regression model included the following variables: age, education, occupation, primary source of information. The level of significance was set at .05.

### 2.3. Ethical approval

Ethical approval for conducting this study was obtained from Jazan University’s Scientific Research Ethics Committee (REC) (reference number; REC-44/05/401, date December 07, 2022). Consents were taken from all participants prior to participation in the study.

## 3. Results

A total of 599 participants were included in the study. Table 1 shows the sociodemographic profile of the participants. The minimum participants age was 18, the maximum was 71, and the mean was 32.49 ± 10.49. As for the gender, 215 (35.9%) of the participants were males, while 384 (64.1%) were females. As for the education level, 18 (3%) had an education less than high school, 102 (17%) had a high school education, 462 (77.1%) had a college (bachelor’s degree), while 17 (2.8%) had a postgraduate degree (master/PhD). As for the occupation, 86 (14.4%) of the participants were non-medical students, 142 (23.7%) were medical students, 220 (36.7%) were employees, and 151 (25.2%) had other occupations.

**Table 1:** Shows the sociodemographic profile of the participants.

Demographical characteristics	n	%
Age		
Minimum	18	
Maximum	71	
Mean	32.49	
Standard deviation	10.49	
Gender		
Male	215	35.90
Female	384	64.10
Education level		
Less than high school	18	3.00
High school	102	17.00
College (bachelor’s degree)	462	77.10
Postgraduate degree (master/PhD)	17	2.80
Occupation		
Non-medical student	86	14.40
Medical student	142	23.70
Employee	220	36.70
Other	151	25.20

Socio-Demographic Profile of The Participants (n = 599).

**Figure 1.**
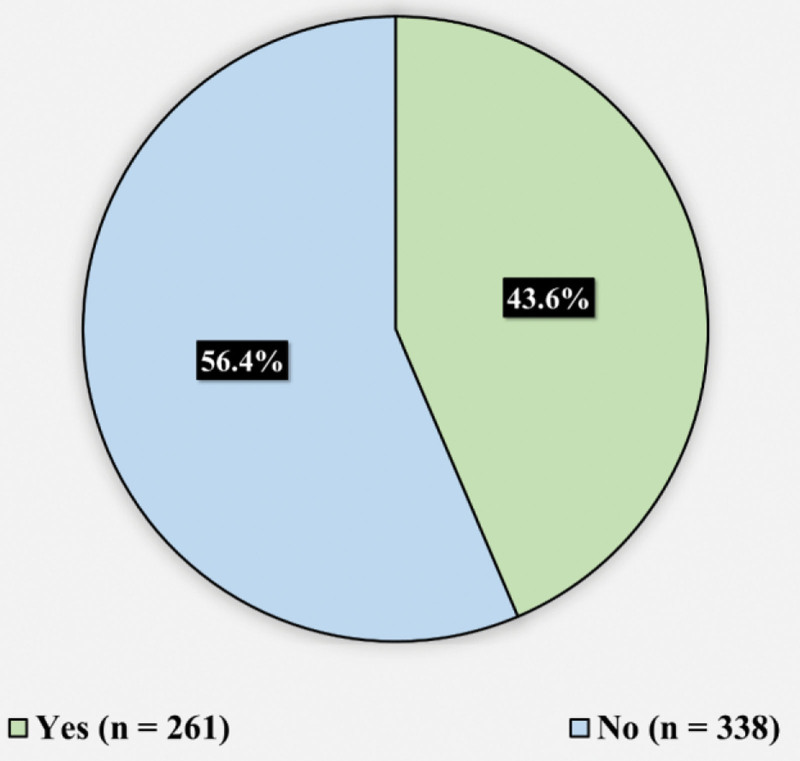
Displays the participants’ responses toward the question “have you previously underwent surgery.

**Figure 2.**
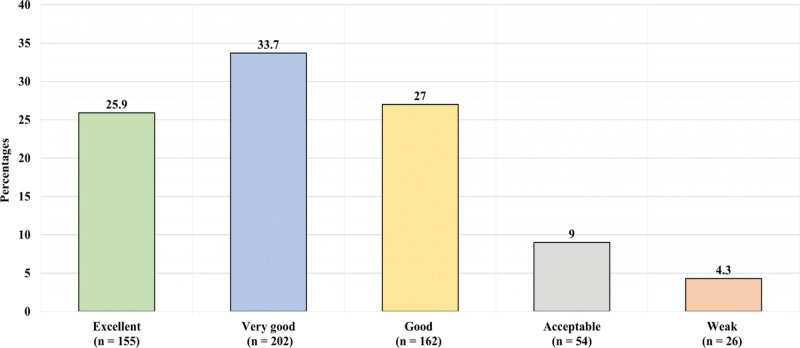
Presents the participants rating toward they knowledge regarding surgical wounds in general.

**Figure 3.**
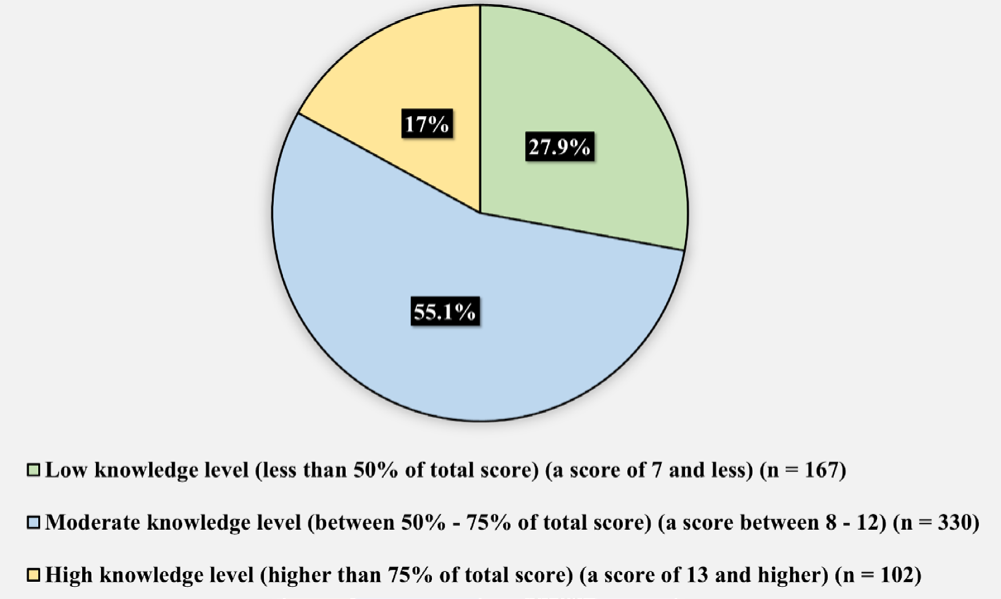
Illustrates the knowledge level toward surgical site infection and wound care.

Figure 1 displays the participants’ responses toward the question “have you previously underwent surgery?” 261 (43.6%) reported previously undergoing surgery, while 338 (56.4%) reported never undergoing a surgery. Figure 2 presents the participants rating toward they knowledge regarding surgical wounds in general. 155 (25.9%) reported thinking they had excellent knowledge, 202 (33.7%) reported thinking they have very good knowledge, 162 (27%) had good knowledge, 54 (9%) had acceptable knowledge, and 26 (4.3%) had weak knowledge. Table 2 demonstrates the assessment of participants knowledge toward surgical site infection and wound care. The minimum knowledge score was 0, the maximum was 16, and the mean was 9.2 ± 3.5. Figure 3 illustrates the knowledge level toward surgical site infection and wound care. 167 (27.9%) of the participants had low knowledge level (less than 50% of total score) (a score of 7 and less), 330 (55.1%) had moderate knowledge level (between 50–75% of total score) (a score between 8–12), and 102 (17%) had high knowledge level (higher than 75% of total score) (a score of 13 and higher) (n = 102).

**Table 2:** Demonstrates the assessment of participants knowledge toward surgical site infection and wound care.

Question	n	%
Q1/What does the term (wound infection) after surgery mean?		
The presence of infection (inflammation) after surgery in another part of the body, not the site of the wound itself.	37	6.2
The presence of infection (inflammation) after surgery, regardless of the location.	37	6.2
The presence of infection (inflammation) after surgery in the area of the wound itself.	445	74.3
I do not know.	80	13.4
Q2/Do you agree with the following statement? (The presence of certain chronic diseases in the patient has a negative impact on the process of healing and healing of wounds after surgical operations).		
Yes	494	82.5
No	43	7.2
I do not know	62	10.4
Q3/In your opinion, which of these conditions have a negative impact on post-operative wound healing? (You can choose more than one answer)		
Cases of liver failure.	155	25.9
Blood cell deficiency diseases.	297	49.6
Heart and respiratory diseases.	119	19.9
Hypertension.	183	30.6
Diabetes.	507	84.6
Use of cortisone.	151	25.2
Radiotherapy treatment.	112	18.7
Immunodeficiency diseases.	361	60.3
Use of anti-inflammatories.	119	19.9
Q4/In your opinion, which of these symptoms may indicate the presence of infection in the wounds after surgical operations? (You can choose more than one answer)		
Headache.	62	10.4
Redness in the wound area.	448	74.8
Swelling in the wound area.	473	79.0
An increase in body temperature.	417	69.6
An unpleasant odor from the wound.	430	71.8
Nausea.	88	14.7
Anorexia.	63	10.5
Bleeding.	171	28.5
The presence of pain.	318	53.1
Yellowing discoloration of the skin or around the eyes.	54	9.0
Itching around the wound.	301	50.3
I do not know.	34	5.7
Q5/In your opinion, which of these fluids (exudates) if they come out of the wound indicate the presence of infection?		
A clear (almost colorless) liquid.	25	4.2
Blood (red liquid) coming out of the wound.	11	1.8
Purulent fluid or pus from the wound.	280	46.7
All the above.	246	41.1
I do not know.	37	6.2
Q6/Do you agree with this statement? (Wound infection may develop into bacteremia, which leads to a deterioration of the condition and may lead to death if it is not treated quickly and effectively)		
Yes	504	84.1
No	19	3.2
I do not know	76	12.7
Knowledge Score (lowest possible score = 0, highest possible score = 16)		
Minimum	0	
Maximum	16	
Mean	9.2	
Standard deviation	3.5	

Assessment of Participants Knowledge toward Surgical Site infection and Wound Care (n = 599).

Table 3 shows the assessment of participants attitude toward surgical site care. 451 (75.3%) of the participants reported thinking that smoking negatively affects healing of wounds after surgery, 466 (77.8%) reported obesity has negative effect, and 504 (84.1%) reported thinking nutritional status can have negative effect. 451 (75.3%) reported agreeing that for wounds to heal properly, they must be allowed to breathe and dry, 580 (96.8%) reported thinking that washing hands before changing or cleaning the wound is important, and 525 (87.6%) reported thinking that the use of antibiotics after the operation is mandatory for wound healing. The most commonly reported topic of importance to be discussed by the surgeons with patients were wound dressing change methods reported by 531 (88.6%), and when the stitches (sutures) are removed reported by 472 (78.8%).

**Table 3:** Shows the assessment of participants attitude toward surgical site care.

Question	n	%
Q1/In your opinion, which of these factors may negatively affect the healing of wounds after surgery?		
Smoking		
Agree	451	75.3
Do not agree	148	24.7
Obesity		
Agree	466	77.8
Do not agree	133	22.2
Nutrition status		
Agree	504	84.1
Do not agree	95	15.9
Q2/Which of these statements do you think is true and which are false?		
For wounds to heal properly, they must be allowed to breathe and dry.		
Agree	451	75.3
Do not agree	148	24.7
Washing hands before changing or cleaning the wound is important.		
Agree	580	96.8
Do not agree	19	3.2
The use of antibiotics after the operation is mandatory for wound healing.		
Agree	525	87.6
Do not agree	74	12.4
Q3/In your opinion, which of the following is recommended to the patient for the surgeon to discuss about it and know about it, whether it was before the operation or before leaving the hospital after the operation? (You can choose more than one answer)		
Wound dressing change method.	531	88.6
When the stitches (sutures) are removed	472	78.8
When you can take a shower after the operation and the precautions during it	460	76.8
Symptoms associated with postoperative wound infection.	388	64.8
When you should exercise after the operation.	361	60.3
The type of eating he recommends as well as the one you stay away from.	338	56.4
Using of traditional remedies to help heal wounds.	108	18.0

Assessment of Participants Attitude toward Surgical Site Care (n = 599).

Table 4 displays the assessment of participants practice toward surgical site care. 333 (55.6%) reported thinking that aromatic odors (such as perfume or incense) negatively affect wound healing, 251 (41.9%) reported thinking that placing natural honey on the wound is useful for wound healing, 263 (43.9%) reported thinking that water and salt have an effective effect in preventing wound infection, 538 (89.8%) reported thinking that if the wound is inflamed, it may have a strange smell, and 340 (56.8%) reported thinking that during the healing of surgical wounds, you should not leave the house. The majority of participants 316 (52.8%) rated the importance of walking after surgery in term of benefit to be 5 out of 5. 330 (55.1%) of the participants reported that the importance of walking after surgeries lies in reducing risk of blood clots. 349 (58.3%) recognized the importance of post-operative breathing exercise. Among those 349 participants, 219 (62.8%) recognized that the importance of breathing exercise after surgery lies in reducing the risk of developing lung infections after the operation.

**Table 4:** Displays the assessment of participants practice toward surgical site care.

Question	n	%
Q1/What do you think of the following behaviors and practices:		
Aromatic odors (such as perfume or incense) negatively affect wound healing.		
Agree	333	55.6
I do not agree	266	44.4
Placing natural honey on the wound is useful for wound healing.		
Agree	251	41.9
I do not agree	348	58.1
Water and salt have an effective effect in preventing wound infection.		
Agree	263	43.9
I do not agree	336	56.1
If the wound is inflamed, it may have a strange smell.		
Agree	538	89.8
I do not agree	61	10.2
During the healing of surgical wounds, you should not leave the house.		
Agree	340	56.8
I do not agree	259	43.2
Q2/In your opinion, how important is walking after surgery in terms of its benefits? (Scoring range from 1 to 5 which is 1 has no benefits and 5 has more benefits).		
1	62	10.4
2	39	6.5
3	106	17.7
4	76	12.7
5	316	52.8
Q3/If you believe that walking after surgery is useful and important, what is it?		
To reduce the risk of blood clots.	330	55.1
To help reduce weight after the operation.	22	3.7
To reduce the complications of the operation.	82	13.7
Because doctors always recommend walking.	70	11.7
To get rid of excess salt.	18	3.0
It has no significance with regard to surgical operations.	20	3.3
I do not know.	57	9.5
Q3/Do you think post-operative breathing exercises have importance that associated with the post-operative phase?		
Yes	349	58.3
No	42	7.0
I do not know.	208	34.7
Q4/If you answered yes to the previous question, why are breathing exercises important after surgery? (n = 349)		
Because the attending physician advised that.	84	24.1
To reduce the risk of developing lung infections after the operation.	219	62.8
To avoid affecting the voice and trachea.	17	4.9
Important only in rib cage operations.	29	8.3

Assessment of Participants Practice toward Surgical Site Care (n = 599).

**Figure 4.**
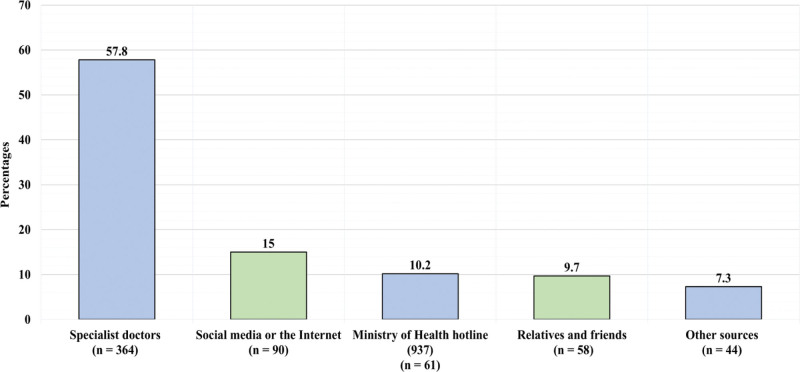
Presents the participants’ source of information toward surgical site infection and wound care.

Figure 4 presents the participants’ source of information toward surgical site infection and wound care. The primary source of information for 364 (57.8%) participants was specialist doctors, for 90 (15%) participants it was social media or the internet, for 61 (10.2%) it was ministry of health hotline, for 58 (9.7%) it was relatives and friends, and for 44 (7.3%) it was others. Table 5 demonstrates the factors associated with knowledge toward surgical site infection and wound care. Education level was significantly associated with knowledge score toward surgical site infection and wound care (*P* = .007), where it was observed that participants with college education had the highest knowledge score. Tukey post hoc test revealed that participants with college education had a significantly higher knowledge score compared to participant with high school education (*P* < .05). Occupation was also significantly associated with knowledge score toward surgical site infection and wound care (*P* < .001), where it was observed that medical students had the highest knowledge score. Tukey post hoc test revealed that medical students had a significantly higher knowledge score compared to both non-medical students, and employee respectively (*P* < .05). Primary source of information was also significantly associated with knowledge score toward surgical site infection and wound care (*P* = .021), where it was observed that those with specialist doctors as the primary source of information had the highest knowledge score. Tukey post hoc test revealed that those with specialist doctors as the primary source of information had a significantly higher knowledge score compared to those with social media and internet as the primary source of information (*P* < .05). Age had significant weak negative correlation with knowledge score toward surgical site infection and wound care (*P* < .001, correlation coefficient = - 0.18).

**Table 5:** Demonstrates the factors associated with knowledge toward surgical site infection and wound care.

Factor	Knowledge score		*P* value
	Mean	Score	
Gender			.112
Male	8.89	3.70	
Female	9.37	3.44	
Education level			.007*
Less than high school	8.39	3.55	
High school	8.29	3.98	
College (bachelor’s degree)	9.47	3.38	
Postgraduate (master/PhD)	8.12	4.00	
Occupation			<.001*
Non-medical student	8.27	3.45	
Medical student	11.85	2.84	
Employee	8.15	3.23	
Have you previously undergone a surgery?			.439
Yes	9.07	3.22	
No	9.30	3.77	
Participants’ source of information toward surgical site infection and care			.022*
Social media or the Internet.	8.42	3.90	
Relatives and friends	8.86	2.59	
Specialist doctors.	9.58	3.44	
Ministry of Health hotline (937)	8.98	2.97	
Age			
*P* value	<.001*		
Correlation coefficient	−0.18		

* Significant at level .05.

Table 6 illustrates the multivariate logistic regression (factors predicting high knowledge level toward surgical site infection and wound care). The logistic regression model included the following variables: age, education, occupation, primary source of information. The only factor that predicted high knowledge level toward surgical site infection and wound care was being a medical student (*P* < .001, odds ratio = 5.9). Age, education, and primary source of information were not predictors of high knowledge level toward surgical site infection and wound care.

**Table 6:** Illustrates the multivariate logistic regression (factors predicting high knowledge level toward surgical site infection and wound care).

Factor	*P* value	Odds ratio	Confidence interval	
Age	.859	1.01	0.95	1.06
Education (High school vs college (bachelor’s degree))	.85	0.92	0.39	2.15
Occupation (non-medical student is the referent)				
Medical student	<.001*	5.90	2.43	14.33
Employee	.465	0.60	0.15	2.39
What is your primary source of information regarding wound and wound care? (Social media or the internet is the referent)				
Relatives and friends	.464	0.62	0.17	2.25
Specialist doctors.	.596	1.23	0.57	2.66
Ministry of Health hotline (937)	.334	0.53	0.15	1.92

* Significant at level .05.

## 4. Discussion

SSIs refer to infections that compromise either the incision or the deep tissue at the site of operation within 30 days post-surgery, or within 90 days if an implant remains in place after treatment.^[11]^ SSIs continue to pose significant challenges to healthcare systems, causing morbidity, mortality, and resource strain, despite advancements in surgical techniques and infection control methods.^[12,13]^ Our study aimed to evaluate the knowledge, attitudes, and practices of participants concerning SSIs and wound care. We included 599 participants and discovered that medical students exhibited the highest knowledge scores compared to non-medical students and employees. Specialist doctors served as the primary information source on SSIs and wound care. Participants considered handwashing, maintaining a dry environment around the wound, and prophylactic antibiotic use to be crucial for proper wound care. They also believed that factors such as smoking, obesity, and poor nutritional status negatively impacted wound healing. Honey and saline solutions were thought to help prevent wound infections.

In our study, 43.6% of subjects had undergone surgery in their lifetime, a slightly higher percentage than reported in previous studies by Jan et al^[14]^and Magill et al^[15]^ (29.2% and 39.5%, respectively). Moreover, we found that 25.9% of participants had excellent knowledge of wound care, lower than the findings of Woldegioris et al^[16]^ However, this discrepancy in knowledge levels could be attributed to the use of different knowledge scales. Our study’s average SSI knowledge score was roughly fair, aligning with local literature (Alsahli et al; Malaekah et al).^[17,18]^

Various factors, such as smoking, nutritional status, and chronic conditions like diabetes mellitus, can influence wound healing (Brown et al; Guo and Dipietro; Liu et al).^[19–21]^ Our findings indicate that most subjects acknowledged the negative impact of smoking, nutritional status, aromatic odors (e.g., perfumes), and obesity on wound healing. Additionally, they believed that proper handwashing, prophylactic antibiotic use, and fostering a dry environment around the wound positively affected wound healing, consistent with findings of a relevant article conducted among nurses (Woldegioris et al).^[16]^ Almost half of the patients thought that applying natural honey to the wound could enhance the healing process, this finding might be supported by a 2015 review article outlining the benefits of topical honey treatment on wound sites (Jull et al).^[22]^ Similarly, to Malaekah et al^[18]^ we found that half of the participants believed that saline solution could help prevent SSIs.

Participants were most concerned about proper dressing changes and suture removal after surgery. In contrast, a study by Malaekah et al^[18]^ among parents found that SSIs were the primary concern. The majority of subjects (57.8%) in our study preferred receiving wound-healing information from healthcare professionals, doubling the percentage reported by Malaekah et al^[18]^ Furthermore, in our study, we found this group demonstrated the highest knowledge level compared to others (*P* = .002).

Educational level played a significant role in participants’ knowledge levels (*P* = .007), with those holding college degrees scoring significantly higher than those with high school diplomas or lower qualifications, paralleling previous study findings by (Priyadarshani and Samarawickrama).^[23]^ Among college-educated individuals, medical students scored the highest (*P* < .001), and this remained a significant predictor in adjusted multivariate analysis. Interestingly, we found a significant inverse relationship (*P* < .001) between age and knowledge level, contrasting with the findings of Priyadarshani and Samarawickrama.^[23]^ We note that the reason for this difference may be due to the lack of sample in the other study in addition to the method of analysis.

In the current study, the most important source of information about surgical site infection was specialized doctors with a percentage of 57.8%, followed by social networking sites with a percentage of 15%, followed by the Ministry of Health hotline with a percentage of 10%. It is consistent with another study that was established in Asir in 2020, where it was found Most of the information was about health practitioners, followed by friends, then the Ministry of Health hotline, and then social networking sites.^[14]^

Our study has a few limitations that we want to be considered during the interpretation of the results. Firstly, we conducted the study through an online survey using a convenience sampling method. Therefore, the sample won’t be representative of the overall population, especially elderly patients. Secondly, since the participants self-administered their awareness level, there may be a possibility of bias. Thirdly, we did not observe the actual wound care practices or outcomes but relied on the participants’ self-administration. Therefore, the study findings may not accurately reflect real wound care practices or outcomes.

Despite those limitations, our study provided insights into the current knowledge, attitudes, and practices of the participants regarding SSI and wound care. The findings indicate areas for improvement in SSI education and awareness-raising, which may aid in developing specific measures that boost wound care processes and outcomes.

In conclusion, our study highlights the need for ongoing education and awareness regarding SSIs and wound care among healthcare professionals and patients. Medical students exhibited the highest knowledge scores, emphasizing the importance of incorporating SSI and wound care education into medical curricula. Healthcare professionals should prioritize patient education on proper wound care practices, including hand hygiene, maintaining a dry environment, and prophylactic antibiotic use. Future research should focus on evaluating the effectiveness of educational interventions on improving SSI and wound care knowledge and practices.

## Acknowledgments

The authors are immensely thankful to the data collectors and participants of this study.

## Author Contributions

**Conceptualization:** Hassan Mashbari, Sulaiman Hamdi, Hussam Darraj, Mohammed Awaf, Shaden Zaalah, Faisal Hakami, Khalid M. Hakami, Essam Alhazmi, Layla Al khairat, Shatha A. Hakami, Amani Aburasain, Ibrahim Ali I. Hakami, Abdulaziz A. Arishi.

**Data curation:** Sulaiman Hamdi, Hussam Darraj, Mohammed Awaf, Shaden Zaalah, Faisal Hakami, Khalid M. Hakami, Essam Alhazmi, Layla Al khairat, Shatha A. Hakami, Amani Aburasain.

**Formal analysis:** Hassan Mashbari, Ibrahim Ali I. Hakami, Abdulaziz A. Arishi.

**Investigation:** Hassan Mashbari, Sulaiman Hamdi, Ibrahim Ali I. Hakami, Abdulaziz A. Arishi.

**Methodology:** Hassan Mashbari, Hussam Darraj, Mohammed Awaf, Shaden Zaalah, Faisal Hakami, Khalid M. Hakami, Essam Alhazmi, Layla Al khairat, Shatha A. Hakami, Amani Aburasain.

**Project administration:** Hassan Mashbari, Ibrahim Ali I. Hakami, Abdulaziz A. Arishi.

**Resources:** Hassan Mashbari.

**Software:** Hassan Mashbari.

**Supervision:** Hassan Mashbari.

**Validation:** Hassan Mashbari.

**Writing – original draft:** Hassan Mashbari, Sulaiman Hamdi, Hussam Darraj, Mohammed Awaf, Shaden Zaalah, Faisal Hakami, Khalid M. Hakami, Essam Alhazmi, Layla Al khairat, Shatha A. Hakami, Amani Aburasain.

**Writing – review & editing:** Hassan Mashbari, Ibrahim Ali I. Hakami, Abdulaziz A. Arishi.
